# Uptake of Phosphorus from an Acidic Kraft Pulp Industrial Effluent Using Magnetic Nanoparticles

**DOI:** 10.1021/acssusresmgt.4c00346

**Published:** 2025-01-25

**Authors:** Celso E. D. Cardoso, Joana C. Almeida, João Rocha, Eduarda Pereira

**Affiliations:** † Department of Chemistry, 56062 CICECO-Aveiro Institute of Materials, University of Aveiro, Campus de Santiago, 3810-193 Aveiro, Portugal; ‡ Department of Chemistry, LAQV-REQUIMTE, University of Aveiro, Campus de Santiago, 3810-193 Aveiro, Portugal

**Keywords:** cobalt ferrite, phosphate, adsorption, eutrophication, regeneration

## Abstract

Global population growth and industrialization have increased the demand for natural resources, notably phosphorus, which is essential for agricultural and industrial applications. However, the scarcity of phosphorus and its environmental ramifications require innovative solutions for its removal and reuse. This study assesses cobalt ferrite nanoparticles’ efficacy in removing phosphorus from kraft pulp effluents. Comprehensive sampling was conducted at various paper pulp facilities utilizing *Eucalyptus globulus* and the kraft pulp process. Variables, such as pH, temperature, sorbent dose, and initial phosphorus concentration, were investigated. Experiments were performed on streams containing 5, 25, and 45 mg/L phosphorus. At 5 mg/L, the nanoparticles achieved up to 93% P removal at pH 6, 60 °C, and 1.0 g/L nanoparticles. Kinetic studies suggested that the adsorption process conforms to the pseudo-second-order model, indicative of chemisorption. For streams with concentrations of 25 and 45 mg/L, the nanoparticles maintained a rapid adsorption process, achieving 96% removal. The Elovich model aptly described the kinetics, reaffirming chemisorption as the predominant mechanism. Comparative analyses revealed that the nanoparticles outperformed Phoslock (a commercial lanthanum phosphorus sorbent), particularly at shorter contact times and at pH values of 3 and 6. Desorption studies yielded optimal results using a binary solution of NaOH (0.1 mol/L) and Ca­(OH)_2_ (1 mol/L), allowing four cycles maintaining high performances. These findings underline the potential of this technology in effluent treatment, where the reusability of nanoparticles offers a cost-effective strategy for environmental remediation and sustainable water and phosphorus management.

## Graphical abstract

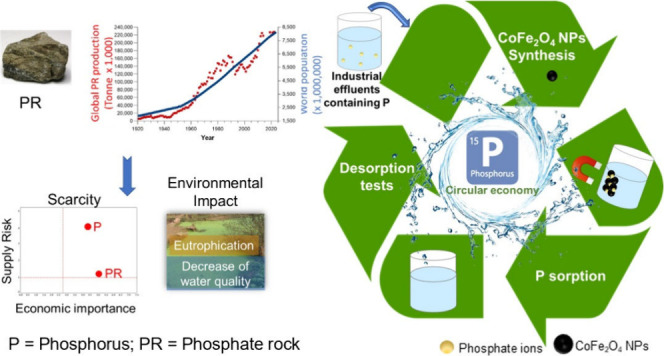

## Introduction

Global phosphorus (P) demand has grown rapidly due to increased agricultural production, with 85% of global reserves found in Morocco (70%), China (5%), Egypt (4%), Algeria (3%), and Syria (3%).^1,2^ The EU is heavily dependent on P imports, making supply vulnerabilities a critical concern. Price fluctuations, geopolitical tensions, and supply disruptions have led to periods of scarcity and price inflation. As a result, P has been recognized as a critical material,^3,4^ and its efficient management has become increasingly urgent, especially in sectors like agriculture and industry. Concerns over P scarcity and price inflation could be alleviated by adopting a circular economy approach that recovers phosphorus from alternative sources.

Urban and industrial wastewaters, despite variable P levels, offer the potential for P removal and recovery. Several industries, including food processing, metallurgical, textile, and paper and pulp industries, generate P-rich effluents. Reported P concentrations range from 5-20 mg/L in domestic effluents to 194–780 mg/L in swine discharges. Even low concentrations, under 20 mg P/L, are viable for removal and recovery due to the large volumes produced by sectors such as domestic, textile, and pulp mills industries.^4,5^ Effluents from pulp and paper industries using the kraft process produce large wastewater volumes (up to 70 m^3^/ton),^6^ with over 90% of the P originating from wood, especially *E. globulus* from the Iberian Peninsula, which has high P content.^7^ Given that P significantly contributes to the degradation of over a third of Europe’s water bodies,^2^ optimizing kraft effluent treatment is essential. The Best Available Techniques for this sector recommend P emission levels of 0.01-0.03 kg/ADt (kilograms per air dry tonne) for bleached kraft pulp mills, except for those processing Eucalyptus, which are subject to levels of 0.02 to 0.11 kg/ADt due to the high P content. To meet these standards, wastewater treatment plants processing 25 to 50 m^3^/ADt must maintain P levels below 1.2 mg/L and 4.4 mg/L, respectively, for conventional and Eucalyptus kraft pulp mills.^7^ However, these standards may be insufficient to prevent eutrophication, as P concentrations exceeding 100 μg/L pose a risk.^8^ As the EU intensifies environmental policies, industries must anticipate stricter regulations and develop efficient methods to reduce nutrient pollution and P dependency.

Current P effluent treatments encompass physical, biological, and chemical methods with chemical precipitation being prevalent. Despite its widespread use, this method often leads to substantial sludge production from insoluble metals precipitation, elevated operational costs, increased effluent salinity, and posed heavy metal contamination risks, potentially hindering subsequent biological treatments.^7,9^ An alternative to traditional chemical precipitation is sorption, which offers high efficiency, regeneration potential, and minimal waste with a vast selection of sorbent materials available. Materials based on La are effective sorbents for P removal,^8^ attributed to the affinity between lanthanides and phosphates. Nanomaterials, known for their large surface area and exceptional retention ability, have been highlighted as sorbents for P removal from aqueous solutions and wastewaters.^5,10,11^ Examples include zero-valent iron (Fe0),^12^ nanoparticles,^13^ and mixtures of iron oxides, titanium oxides, and silicon oxides.^14,15^ Other materials such as hydrated zirconium oxide, manganese oxide, and zinc ferrite have also been utilized.^12,16^ A case in point is magnetic nanomaterials, which hold much potential as adsorbents because their magnetic properties enable separation from water using an external magnetic field, avoiding the use of traditional purification techniques like filtration and sedimentation. In a study conducted by Ajmal et al.,^17^ iron oxide nanoparticles such as ferrihydrite, goethite, and magnetite were examined. These nanoparticles achieved P removal rates of 66.6, 57.8, and 50.5 mg P/g, respectively, when tested with ultrapure solutions in a neutral pH range. Chen et al.^18^ reported a magnetic lanthanum composite, with an adsorption capacity of 104.01 mg P/g from deionized water. Karthikeyan et al.^19^ engineered a magnetic inorganic–organic hybrid composite of porous carbon obtained from papaya seeds, which exhibited a maximum adsorption capacity of 92.34 mg/g phosphate using deionized water as matrix, equivalent to 30.11 mg P/g.

Despite the extensive research on adsorbents and nanoadsorbents for P removal, the real-world applicability of these materials remains largely untested under real wastewater conditions. In most cases, experimental conditions do not represent practical scenarios, with very simple synthetic matrices and high amounts of adsorbent used as referred to above. To the best of our knowledge, the potential of ferrite nanoparticles for P removal or recovery from industrial effluents, specifically those originating from pulp and paper mills, has not been assessed. The present study explores the potential of these materials for P removal and recovery from streams of *E. globulus* kraft pulp production facilities. In particular, cobalt ferrite nanoparticles have been prepared, and their effectiveness in adsorbing P from the D_0_ streams of three distinct Portuguese kraft pulp facilities has been assessed. These streams correspond to the effluent produced from the initial stage of the bleaching process where chlorine dioxide is used. The key variables of these streams, including pH, temperature, sorbent dose, and initial P concentrations, were thoroughly examined. Three P concentrations (5, 25, and 45 mg/L) were selected to encompass the typical range of P concentrations found in this process stream. The study also considered two pH values (the effluent’s original pH of around 3 and an adjusted pH of 6, closer to the standard discharge pH requirements) and three temperatures (60, 40, and 20 °C), reflecting a range from the effluent’s initial temperature to room conditions. This comprehensive analysis aims to provide a deeper understanding of the factors influencing P adsorption and to optimize the conditions for maximum P removal. Removing P from these streams has the potential to mitigate the threats of eutrophication, improve water quality, and prevent pulp production issues such as pipe blockages due to phosphate salt precipitation.

## Experimental Section

### Materials and Reagents

All reagents used in this study were obtained from certified suppliers and used without additional purification. These included potassium hydroxide (KOH, >98%), potassium nitrate (KNO_3_, >99%), ferrous sulfate heptahydrate (FeSO_4_·7H_2_O, >99%), cobalt chloride hexahydrate (CoCl_2_·6H_2_O, >98%), potassium dihydrogen phosphate (KH_2_PO_4_, >99%), and sodium hydroxide (NaOH, >98%) that were obtained from Chem-lab NV. The ultrapure water (18 MΩ cm) used was generated by a Millipore Integral 10 system. Prior to the experiments, all glassware was cleaned with nitric acid (HNO_3_ 25% v/v), sourced from Merck, Suprapur® 65%, for a minimum of 24 h, followed by rinsing with ultrapure water (Milli-Q water, 18 MΩ/cm). Sorption experiments were conducted using acidic P-rich streams derived from a pulp mill. This effluent, also known as the “D_0_ stream”, corresponds to the filtrate from the pulp washing obtained at the termination of the D_0_ stage (the initial chlorine dioxide stage) of a bleaching sequence employed at a pulp and paper production facility utilizing the Kraft process. The temperature of this effluent hovers around 60 °C.

### Synthesis, Structural, and Chemical Characterization of the Cobalt Ferrite Nanoparticles

Cobalt ferrite nanoparticles with an average size of 50 nm were synthesized via oxidative hydrolysis of iron­(II) sulfate under alkaline conditions, scaling-up the method described by Tavares et al.^20^ Briefly, 15.20 g of KOH and 12.16 g of KNO_3_ were added to 200 mL of deoxygenated water in a round flask. This mixture was then heated at 60 °C under a nitrogen atmosphere while being mechanically stirred at 500 rpm. Once fully dissolved, 200 mL of an aqueous solution containing 24.56 g of FeSO_4_·7H_2_O and 11.52 g of CoCl_2_·6H_2_O was added dropwise to the mixture while increasing the stirring speed to 700 rpm. The solution was allowed to react for 30 min, and then the round flask was moved to a hot oil bath maintained at 90 °C, under a nitrogen atmosphere, without stirring, for 4 h. Finally, the obtained dark brown powder was washed and dried at 60 °C.

A variety of techniques were employed to perform the physical and chemical characterization of cobalt ferrite nanoparticles. The morphology of these nanoparticles was examined using Transmission Electron Microscopy (TEM) with an Electron Microscope JEOL 2200FS operating at 200 kV. The zeta potential measurements of the colloidal samples were conducted using a Zetasizer Nano ZS from Malvern Instruments. The pH of the colloid was adjusted between 2 and 10 using either NaOH or HNO_3_ aqueous solutions. The measurements were carried out at a constant temperature of 25 °C, with each sample measured three times for accuracy. The crystalline phase present in the powdered sample was determined using X-ray Diffraction (XRD) on an Empyrean PANalytical diffractometer, utilizing Cu Kα_1,2_ X-radiation (λ_1_ = 1.54060 Å; λ_2_ = 1.54443 Å). The diffraction patterns were recorded in continuous mode with a step size of 0.026°, covering the 15 ≤ 2θ ≤ 95° range. Fourier Transform Infrared (FT-IR) spectra were obtained in the range 3800-400 cm^–1^ using a Bruker Tensor 27 spectrophotometer. The instrument was equipped with an Attenuated Total Reflectance (ATR) accessory, and the spectra were recorded after 256 scans at a resolution of 4 cm^–1^.

### Sampling at Kraft Pulp Production Plants

Comprehensive sampling was conducted at various paper pulp facilities using *E. globulus* and the kraft pulp process. For this purpose, different liquid samples were collected to identify the main sources of phosphorus in the kraft pulp process. These samples included the green liquor, white liquor, D_0_ stream, acid effluent, effluent at the primary treatment inlet (EPTI), effluent at the biological treatment inlet (EBTI), and final effluent. The samples were then divided into dissolved fractions and total suspended solids (TSSs). The characterization involved measuring the pH and conductivity, determining TSS, and chemically quantifying total phosphorus (TP).

### Phosphorus Quantification

The quantification of P in solution was carried out using Inductively Coupled Plasma-Optical Emission Spectometry (ICP-OES) employing a Horiba Jobin Yvon Activa M spectrometer (radial configuration) equipped with a Burgener MiraMist nebulizer. Calibration curves were constructed using five standards with concentrations ranging from 0.1 to 45 mg/L. These were prepared by diluting P commercial certified stock solutions in water acidified with 1% HNO_3_ (v/v). Calibration curves were only accepted if they had a correlation coefficient greater than 0.9995. The limit of quantification was set at the lowest standard of the calibration curve (0.1 mg/L). The variation between sample replicates (a minimum of three replicates were examined) was kept below 5%.

The TSSs were subjected to an acid digestion procedure, and the resulting solution was also analyzed for P using ICP-OES.

The quantity of P that the sorbent material retained per unit mass, denoted as q (mg/g), was calculated considering that all of the P extracted from the solution was bound to the sorbent. This was determined using Equation 1

1 $$q=\frac{\left(C_{0}-C_{t}\right)}{m}\times V$$

where C_0_ (mg/L) is the initial concentration of P in solution, C_*t*_ (mg/L) is the concentration of P at time *t*, m is the mass of sorbent (g), and *V* is the solution volume (L).

The removal efficiency, R (%), for P in cobalt ferrite NPs was calculated as follows (Equation 2):

2 $$\mathrm{Removal}\;\left(\%\right)=\frac{\left(C_{0}-C_{t}\right)}{C_{0}}\times 100$$

### Sorption Experiments

Batch sorption trials were carried out to evaluate the effectiveness of cobalt ferrite nanoparticles to remove P from the stream of pulp mills. The acidic D_0_ stream from kraft pulp mills typically has a pH around 3 and a temperature of 60 °C. A range of conditions was tested in these experiments, including varying pH, quantities of sorbent, temperatures, and initial concentrations of P. The experimental parameters were evaluated in streams with low (5 mg P/L), intermediate (25 mg P/L), and high P concentration (45 mg P/L), due to the large range of P concentration find in this industry. Alongside these, control tests were also conducted, consisting of the matrix under study in the absence of cobalt ferrite NPs. All the experiments were performed utilizing Schott glass bottles with a capacity of 100 mL, stirred at 300 rpm in an orbital incubator shaker. The sorption experiments from a low P concentration stream containing 5 mg P/L were conducted with 0.5 and 1.0 g/L NPs, at pH 3 and 6, and at a temperature of 20, 40, and 60 °C. Adjustments of pH were performed using nitric acid or sodium hydroxide solutions. Samples were taken at 15 min, 1 and 24 h, placed under the influence of an external magnet and later analyzed for P. Subsequently, for the sorption kinetic evaluation, 1 g/L cobalt ferrite NPs were added to the D_0_ stream with 5 mg P/L at pH 6 and then placed under stirring at 300 rpm and 60 °C for 48 h. Assessment of experimental parameters was also performed in an acidic D_0_ stream with intermediate and high P concentrations, containing 25 and 45 mg P/L, respectively. These experiments were conducted with 1.0, 3.0, and 5.0 g/L NPs, at pH 3, 6 and 9, and at a temperature of 20, 40, and 60 °C. The samples separation and analysis were carried out as described above. The sorption kinetics were performed for 48 h on the D_0_ stream with 25 mg of P/L at pH 6 and 60 °C using 2 g/L cobalt ferrite NPs. The performance of cobalt ferrite nanoparticles for the removal of P was compared with the commercial sorbent Phoslock. The comparison with the Phoslock was carried out on the D_0_ stream with 25 mg of P/L using 2 g/L sorbent. The temperature in these assays was maintained at 60 °C, and the pH was adjusted to 3 and 6.

### Regeneration and Reusability of Cobalt Ferrite Nanoparticles

In the regeneration studies, 2 g/L nanoparticles were loaded with P aqueous solution using an initial concentration of 25 mg/L at pH 6 over a period of 2 h. Following sorption, the nanoparticles were magnetically separated, subjected to a rinse with ultrapure water, and subsequently dried. The desorption of P from the nanoparticles was then assessed using various concentrations of nitric acid (1 and 2%), hydrochloric acid (0.037, 0.37 and 1.85%), and sodium hydroxide (0.01, 0.1, and 1 mol/L) over durations of 0.25 and 1 h. Following the desorption process, the nanoparticles were isolated magnetically, cleansed with ultrapure water, and dried. The reusability of the sorbent was then assessed through a second sorption experiment. Additional studies were conducted with binary solutions to regenerate the nanoparticles using a mixture of two solvents, Solvent A (1 mol/L) and Solvent B (0.1 mol/L) over a period of 2 h. Sodium hydroxide was selected as Solvent A, while Solvent B varied among CaCl_2_, CaO, CaSO_4_, and Ca­(OH)_2_. The nanoparticles were subjected to four cycles of reuse, and this experiment was repeated 3 times to validate the results.

## Results and Discussion

### Characterization of the Cobalt Ferrite Nanoparticles

Cobalt ferrite (CoFe_2_O_4_) nanoparticles were produced through the chemical oxidative hydrolysis process of Fe­(III) and M­(II) mixtures under alkaline conditions. The crystalline phases and the spinal ferrite structure of the nanoparticles were validated through powder XRD analysis (Figure 1aSI in the Supporting Information).^21^ A representative TEM image of the CoFe_2_O_4_ nanoparticles (Figure 1bSI in the Supporting Information) reveals a particle size distribution ranging from 47 to 99 nm, with an average size of 73 nm. The zeta potential of these nanoparticles was observed to decrease with an increase in solution pH (Figure 1cSI in the Supporting Information), and the isoelectric point was determined to be 6.3. The FTIR spectrum of cobalt ferrite nanoparticles (Figure 1dSI, Supporting Information) exhibits a broad band in the range of 3642-2860 cm^–1^, corresponding to the vibrations of ν­(OH) and the combined vibration δ­(H–O–H) at 1632 cm^–1^. This suggests the presence of molecular water adhering to the surface or its integration within the crystalline lattice.^22,23^ Additionally, the band observed at 1105 cm^–1^ is indicative of metal–OH and metal–OH_2_ bonds,^24^ implying water sorption on the oxide. Finally, the 564 cm^–1^ band is associated with the metal–oxygen stretching vibration.

### Phosphorus Concentrations

The analysis conducted on the samples from the kraft pulp production process revealed the process streams with the highest P content (Figure 2SI in the Supporting Information). These include the green liquor (72 to 98 mg P/L), white liquor (20 to 50 mg P/L), D_0_ effluent (5 to 50 mg P/L), acid effluent (3 to 44 mg P/L), effluent at the primary treatment inlet (EPTI, 5 to 25 mg/L), effluent at the biological treatment inlet (EBTI, 4 to 14 mg/L), and the final effluent (2 to 9 mg P/L). Total suspended solids (TSSs) contained only minor amounts of P, with the dissolved fraction contributing the most significantly to the total P content. The green liquor, primarily composed of Na_2_CO_3_, Na_2_S, and NaOH, has a pH of 13, TSSs between 1000-1200 mg/L, and a conductivity of 140-165 mS/cm. Despite the green liquor having the highest P content among the process streams, its extreme physicochemical characteristics prevent its use for the removal and recovery of P by sorption. Consequently, the D_0_ stream was selected for this research. This stream is obtained at the termination of the D_0_ stage (the initial chlorine dioxide stage) of the bleaching sequence of a kraft process. It has a pH between 2.0-2.7, TSSs between 370 and 415 mg/L, and a conductivity between 6.4-8.1 mS/cm. Another consideration for selecting this effluent was the sample volume. The D_0_ effluent, following the green liquor, has the smallest volume, as it is at an early stage in the process, thereby simplifying its treatment.

### Phosphorus Sorption Using Cobalt Ferrite Nanoparticles

#### Influence of pH, Sorbent Dose, and Temperature

Effluents with a P concentration of 5 mg/L are typically classified as low-P effluents. Despite this, the large volume of effluents generated by this industry renders it an attractive source for phosphorus recovery. As depicted in Figure 1, a series of experiments were undertaken. The results demonstrated a significant enhancement in P removal when the pH of the solution was raised from 3 to 6. This improvement aligns with findings in the literature, where studies such as those by Gu et al.^25^ demonstrated that zinc ferrite nanoparticles doped with cerium exhibit pH-dependent adsorption, with optimal P removal occurring between pH 5.8 and 7.8. At both acidic and alkaline conditions, P removal decreased significantly, which is consistent with our observations that the sorption efficiency improves at more neutral pH values. The observed decrease in sorption over extended periods at pH 3 suggests that the nanoparticles may not maintain stability at this pH, eventually leading to leaching of iron and/or cobalt. Therefore, adjusting the pH of the stream to more neutral values proves beneficial for sorption efficiency. Regarding the sorbent dose, doubling the amount results in an increase of approximately 12-24% in sorption across different pH and temperatures values. This can be explained since the number of active sites for sorption increases with the increase of sorbent amount. A similar trend was observed by Khamis et al.,^26^ who reported approximately a 15% increase in phosphorus removal when the ZnO NPs dose was increased from 0.015 g/L to 0.03 g/L. This increase can be attributed to the higher number of active sites available for adsorption as the sorbent dose rises. The initial increase in removal efficiency is due to the rapid increase in the available surface area on the sorbent, which enhances the uptake of P ions. However, the adsorption efficiency tends to plateau after a certain dose, indicating a saturation point, beyond which additional increases in dose yield progressively smaller improvements in efficiency. As the temperature rises, so does the sorption, which is in accordance with previous works (Ajmal et al., 2018). This behavior suggests that the adsorption of P onto the NPs is an endothermic process, which has been reported by several research studies,^27,28^ augments the adsorption capacity of the NPs and facilitates the diffusion of unabsorbed P ions onto the NPs.^17^ The most effective P removal in this experiment (93%) was achieved at pH 6, 60 °C, and using a dosage of 1.0 g/L NPs. However, the adsorption capacity is dependent on the sorbent dose, being higher at lower doses. Thus, similar to P removal, the best sorption capacity (8.1 mg/g) was achieved at pH 6 and 60 °C but with a reduced NP dosage of 0.5 g/L.

**Figure 1:**
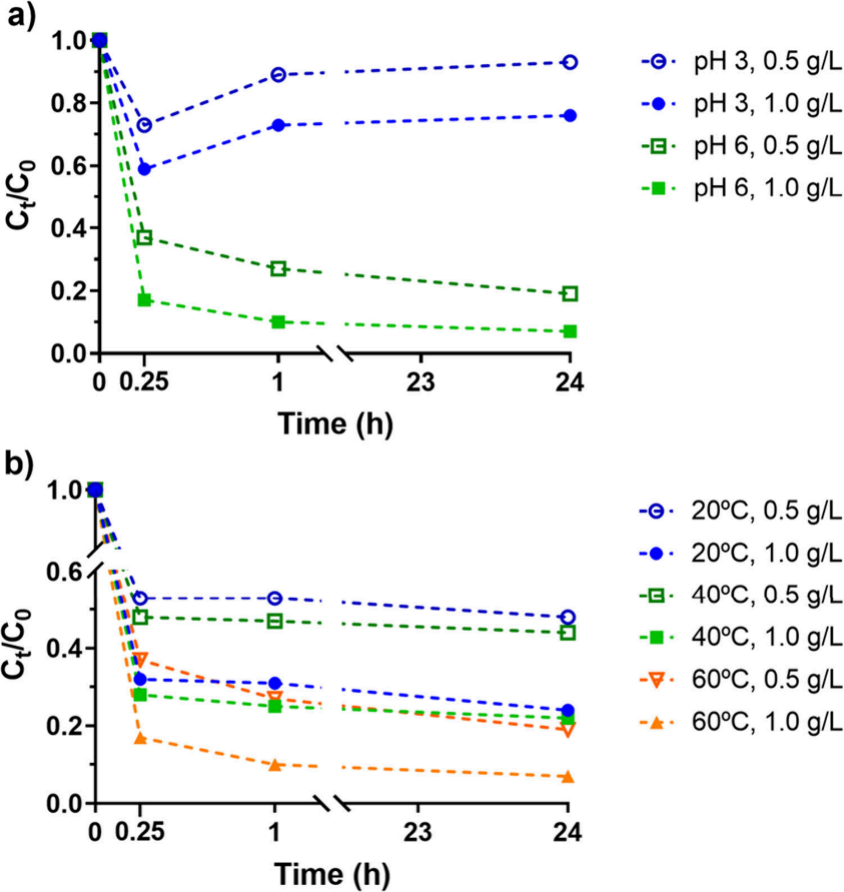
Variation of the normalized P concentrations over time for different experimental parameters using CoFe_2_O_4_ nanoparticles for an initial P concentration of 5 mg/L: a) Effect of pH (3 and 6) and sorbent dose (0.5 and 1.0 g/L) at 60 °C; b) Effect of temperature (20, 40, and 60 °C) and different sorbent doses (0.5 and 1.0 g/L) at pH 6.

The influence of pH, sorbent dose, and temperature on an acidic process stream from a kraft pulp production facility with an initial P concentration of 25 mg/L was evaluated for two pH levels (pH 3 as the baseline and pH 6 as a more neutral condition), three sorbent doses (1, 3, and 5 g/L NPs), and three temperatures (20, 40, and 60 °C) and studied. The findings (Figure 2) indicate that pH ceases to significantly impact the NPs efficiency in the D_0_ stream with 25 mg P/L. Concerning sorbent dose, employing 5 g/L NPs yielded the highest sorption percentages, ranging from 92 to 96%, and adsorption capacities between 4.6 and 4.8 mg/g. Conversely, the use of 3 g/L NPs resulted in sorption percentages between 69 and 86%, with adsorption capacities ranging from 5.8 to 7.2 mg/g. Notably, the differences between the 3 and 5 g/L NP doses decrease with the time of contact. Regarding the temperature effect, no significant differences were observed among the 20, 40, and 60 °C under the experimental conditions evaluated. The diminished influence of temperature on this D_0_ stream, with an initial P concentration of 25 mg P/L, could be attributed to the sorbent dose employed, since the sorbent dose is the experimental parameter that exerts the most pronounced effect on the sorption of P.

**Figure 2:**
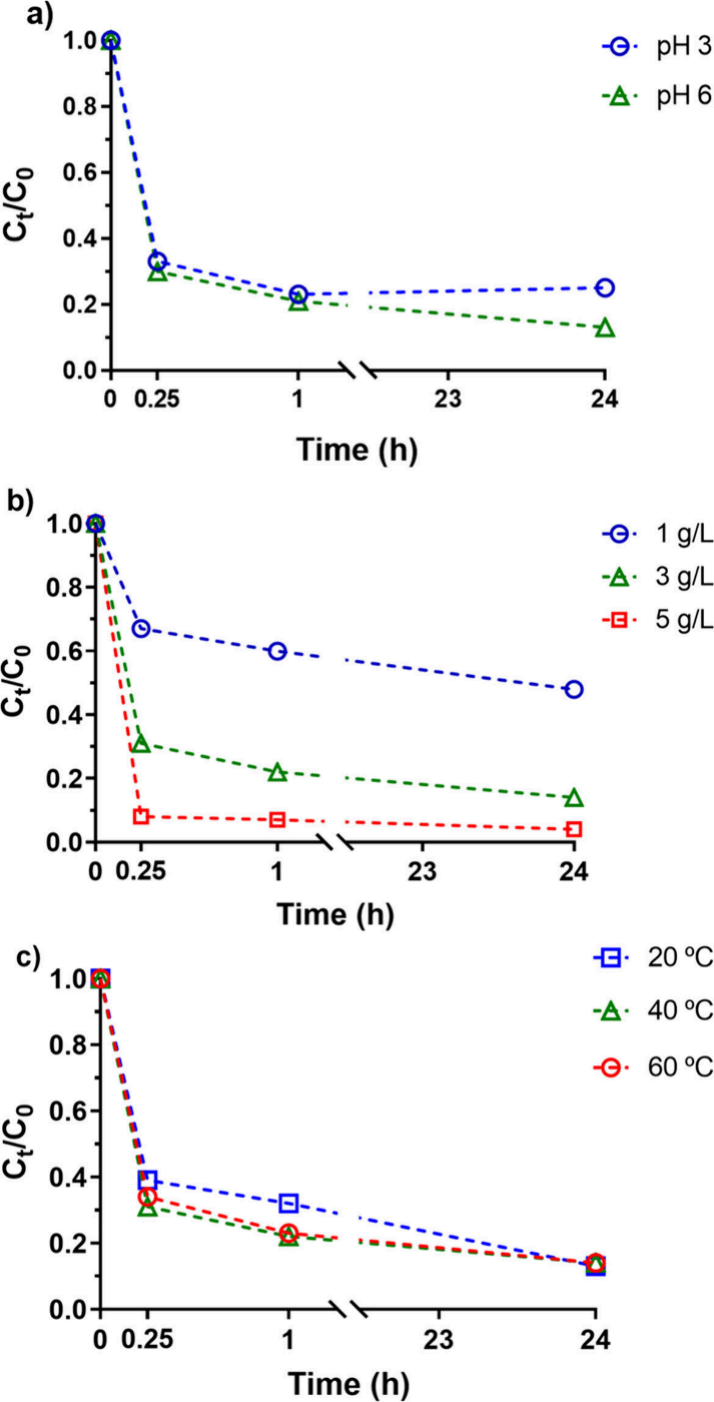
Variation of the normalized P concentrations over time for different experimental parameters using CoFe_2_O_4_ nanoparticles for an initial P concentration of 25 mg/L: a) Effect of pH (3 and 6) with 3.0 g/L NPs at 60 °C, b) Effect of sorbent dose (1, 3, and 5 g/L) at pH 6 and 60 °C, and c) Effect of temperature (20, 40, and 60 °C) with 3.0 g/L NPs at pH 6.

A comparative study was also conducted to evaluate the efficiency of P adsorption using cobalt ferrite NPs and Phoslock, a commercially available material for P removal. The results (Figure 3SI in the Supporting Information) demonstrated that cobalt ferrite NPs outperformed Phoslock in achieving higher P removals, particularly at short contact times and at both pH levels 3 and 6.

The influence of pH and sorbent dose on the efficiency of nanoparticles for P removal from streams with high 45 mg of P/L parallels those observed in streams with 25 mg of P/L (Figure 4SI in the Supporting Information). Increasing the pH does not provide an advantage, as it does not significantly influence sorption. When considering the sorbent dose, at either pH 3 or 6, using 5 g/L NPs instead of 3 g/L improves P removal by 11 to 18% during shorter contact times. In contrast to the experiments conducted in a stream with 25 mg P/L, where only slight differences were observed in sorption at 20 °C or 60 °C for shorter contact times, the differences in streams with 45 mg P/L are more pronounced but decrease over time. The optimal results, with P removals ranging from 80 to 96% over a period of 0.25 to 24 h, were achieved using 5 g/L NPs in a D_0_ stream with a 45 mg P/L at 60 °C and at both pH levels 3 and 6. The highest adsorption capacity of 19.8 mg/g was achieved at pH 3, 60 °C, and with a 1.0 g/L NP dosage, highlighting that lower sorbent doses, while yielding slightly lower removal, can result in higher adsorption capacities.

#### Sorption Kinetics and Modeling

To evaluate the sorption kinetics of CoFe_2_O_4_ nanoparticles for the D_0_ stream with 5 mg P/L, a comprehensive kinetic investigation was conducted at 60 °C and pH 6, over 48 h. The pseudo first-order, pseudo second-order, and Elovich models were employed to evaluate which model best describes the removal process of P by the NPs. The outcomes of this study are represented in Figure 3 and Figure 5SI in the Supporting Information. The P sorption kinetics exhibited by the NPs are remarkably rapid, reaching a substantial 71% within a contact time of just 0.5 h. The highest equilibrium concentration of P in the NPs was 4.3 mg/g, with a sorption efficiency of 87%. Since all the kinetic models employed have an equal number of parameters (two), the R^2^ parameter serves as a valid criterion for evaluating and comparing the effectiveness of the various models in fitting the experimental results. The parameters for the kinetic models employed in this study are detailed in Table 1SI in the Supporting Information. Upon examination of the data presented in Table 1SI, it is discerned that the pseudo second-order model provides the most accurate fit to the experimental data. This indicates that the sorption of P by the NPs is likely a chemisorption process. The similarity of this kinetic trend with those reported in the literature further supports the chemisorption mechanism.^17,29,30^ However, it is important to note that while the pseudo-second-order model suggests a predominant chemisorption pathway, the possibility of concurrent sorption mechanisms cannot be discounted, as reported in Ajmal et al.^17^

**Figure 3:**
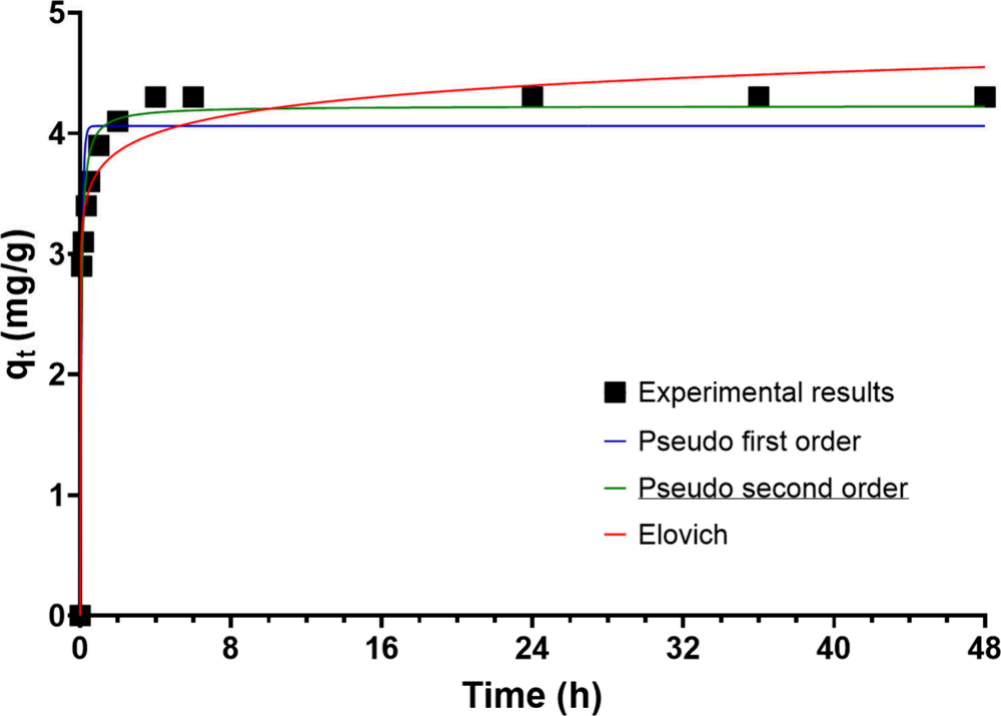
Kinetic models fit (pseudo-first order, pseudo-second order, and Elovich) for P sorption on CoFe_2_O_4_ nanoparticles for the 5 mg P/L stream.

The adsorption kinetics of cobalt ferrite nanoparticles was also evaluated for the D_0_ stream with 25 mg P/L. To this end, 2 g of NPs was added to a SCHOTT flask with 1 L of effluent at pH 6. The solution was maintained at 60 °C during the experiment, and aliquots were collected at different contact times. The removal process of P by the NPs was analyzed using three different models: pseudo first-order, pseudo second-order, and Elovich (Figures 4 and 6SI). The NPs demonstrated a rapid P sorption kinetics, achieving 70% after 3 h of contact. The maximum P equilibrium concentration in the NPs was 9.2 mg/g with a sorption efficiency of 77%. The parameters for the kinetic models employed in this study are detailed in Table 2SI in the Supporting Information. The Elovich model provides the most accurate fit to the experimental data, indicating that the NP’s P sorption is likely a chemisorption process. The Elovich model provides the most accurate fit to the experimental data, indicating that the NP’s P sorption is likely a chemisorption process. This model considers how much P is initially present, the surface area of the NPs, and how they interact with P, showing how these factors affect the availability of binding sites for P on the NPs over time and the energy required for P to attach. This model is useful because it can be applied to different types of adsorbing surfaces and even describes how solutes spread in water.^31,32^

**Figure 4:**
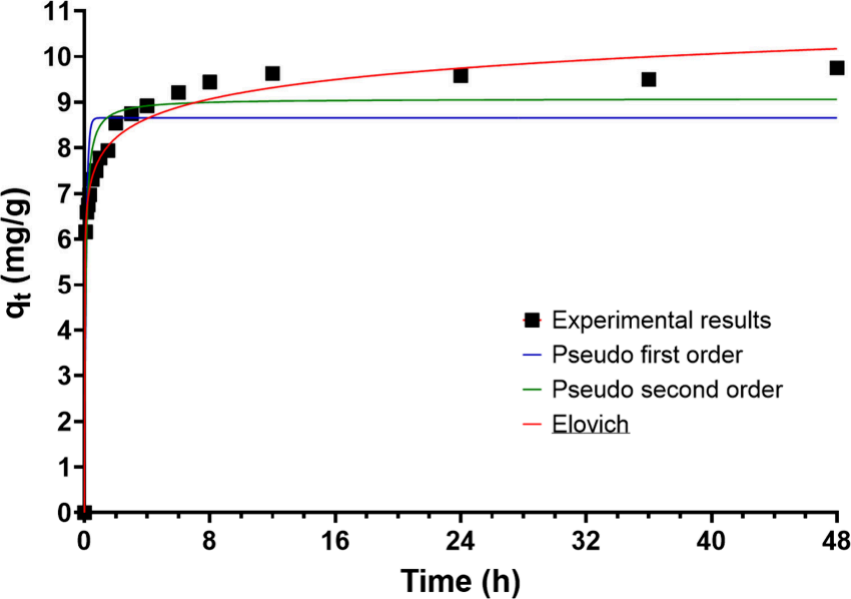
Kinetic models fit (pseudo-first order, pseudo-second order, and Elovich) for P sorption on CoFe_2_O_4_ nanoparticles for the 25 mg P/L stream.

#### Comparison with the Literature

The cobalt ferrite nanoparticles exhibited rapid kinetics and achieved significant P removal across various conditions in very complex matrices such as those from the kraft pulp process. While direct comparisons with other sorbents are challenging due to the unique conditions of this work, we note that Gu et al.^25^ developed zinc ferrite nanoparticles doped with cerium, and their findings indicate a pH-dependent adsorption, with optimal P removal between pH 5.8 and 7.8, decreasing significantly at both acidic and alkaline conditions. The maximum Langmuir adsorption capacity (Qm) increased significantly with cerium doping from 5.2 to 41.6 mg/g. The nanoparticles used in this study, in contrast, show less pH sensitivity and exhibit a higher adsorption capacity than zinc ferrite. Also, Mu et al.^33^ reported a Fe_3_O_4_@Phoslock composite to remove P from ultrapure solutions, demonstrating a removal capacity of 8.1 mg P/g. In a subsequent experiment, the composite was shown to be capable of removing approximately 75% of P from a treated leachate with 3.5 mg of P/L, derived from real sewage. Yuan et al.^34^ reported a magnetic adsorbent (Fe_3_O_4_-doped spent Fluid Catalytic Cracking catalysts), employed to investigate the P removal from a simulated wastewater with 50 mg P/L at pH 7 and using 8 g/L adsorbent capable of removing 6.32 mg P/g. In summary, previous studies have demonstrated the ability to remove P within the first 3 to 5 h of contact time, which aligns with the results of this work. When compared with the existing literature, it is evident that some adsorbents require larger quantities of sorbent for effective P removal. However, the main drawback of the studies reported in the literature is the use of simple matrices. No studies have been found that test the capability of adsorbents to remove P in industrial streams or effluents, particularly those from the pulp and paper industries. Therefore, this study provides valuable insights into the practical application of ferrite nanoparticles in this industry.

#### Regeneration and Reusability of Cobalt Ferrite Nanoparticles

To desorb phosphorus from nanoparticles, various acidic (nitric and hydrochloric acids) and basic (sodium hydroxide) solutions were examined at diverse concentrations, from 0.01 to 0.6 mol/L. The solvents that demonstrated the highest efficacy were hydrochloric acid at a concentration of 0.37% and sodium hydroxide at a concentration of 0.1 mol/L (Figure 7SI in the Supporting Information). Despite the success in desorbing P, it was found that the NPs lost their sorption efficiency, making their application in a second sorption cycle not viable. To address this issue, additional regeneration studies were conducted. Basic binary solutions were used to desorb and regenerate the NPs. The binary solution consisted of a blend of solvent A at a concentration of 1 mol/L and solvent B at a concentration of 0.1 mol/L. The best results were achieved using the binary solution NaOH (0.1 mol/L) + Ca­(OH)_2_ (1 mol/L), followed by NaOH (0.1 mol/L) + CaO (1 mol/L), and NaOH (0.1 mol/L) + CaCl_2_ (1 mol/L). The efficiency of the NPs in P recovery using the optimal binary solution combination of NaOH (0.1 mol/L) and Ca­(OH)_2_ (1 mol/L) was 74, 93, 66, and 73% P across the four cycles. A possible explanation for the increase in P recovery from 74 to 93% between the first and second cycles could be due to the formation of calcium phosphate on the surface of the NPs. Calcium phosphate is a precipitate that can enhance the P sorption capacity of sorbents by providing more active sites for P binding.^26^

These results are highly promising, demonstrating the potential of this technology in treating P-rich streams from pulp and paper mills. The ability to reuse nanoparticles for at least four cycles presents a significant opportunity for cost reduction in effluent treatment using this technology.

## Conclusions

This work highlights the potential of cobalt ferrite nanoparticles as an efficient and sustainable solution for the removal and recovery of phosphorus from kraft pulp effluents. Through meticulous experimentation across various operational conditions, these nanoparticles have shown remarkable phosphorus removal and recovery rates, outperforming a commercial sorbent. The findings also indicate that the adsorption process is predominantly chemisorptive. The ability to remove up to 96% of phosphorus in higher concentration streams and maintain high performance over multiple desorption cycles underscores the practical applicability of cobalt ferrite nanoparticles in industrial settings. The successful use of a binary solution for nanoparticle regeneration presents a cost-effective and environmentally friendly approach to effluent treatment. This technology holds significant promise for enhancing sustainable water and phosphorus management practices, contributing to the broader goal of achieving environmental equilibrium in the midst of intensive industrial activity.

## Supplementary Material



## Notes

Figures of NPs characterization: XRD diffractogram, TEM image, graph of zeta potential as a function of pH, FTIR spectra; P concentration in different samples from a pulp mill facility, comparison of NPs removal efficiency with Phoslock, NPs efficiency for an initial P concentration of 45 mg/L, desorption of P from NPs using acidic and basic solutions, equations and parameters of the kinetic models (PDF)
