# Supplementary material for: Uptake of Phosphorus from an Acidic Kraft Pulp Industrial Effluent Using Magnetic Nanoparticles
Source: ACS Sustain Resour Manag. 2025 Jan 25;2(2):267–74. doi: 10.1021/acssusresmgt.4c00346 (PMC12776358; doi:10.1021/acssusresmgt.4c00346)
Supplement: Supplementary file 1 [file rm4c00346_si_001.pdf]

Supporting information

Uptake of phosphorus from an acidic kraft pulp industrial effluent using magnetic nanoparticles

Celso E. D. Cardoso[†,‡], Joana C. Almeida[†,‡], João Rocha[†], Eduarda Pereira[‡*]

[†]Department of Chemistry, CICECO-Aveiro Institute of Materials, University of Aveiro, Campus de Santiago, 3810-193, Aveiro, Portugal

[‡]Department of Chemistry, LAQV-REQUIMTE, University of Aveiro, Campus de Santiago, 3810-193, Aveiro, Portugal

Corresponding author

*Eduarda Pereira. Tel: +351 234 370 721. E-mail: eduper@ua.pt

Number of pages: 9; Number of figures: 7; Number of tables: 2

Figures

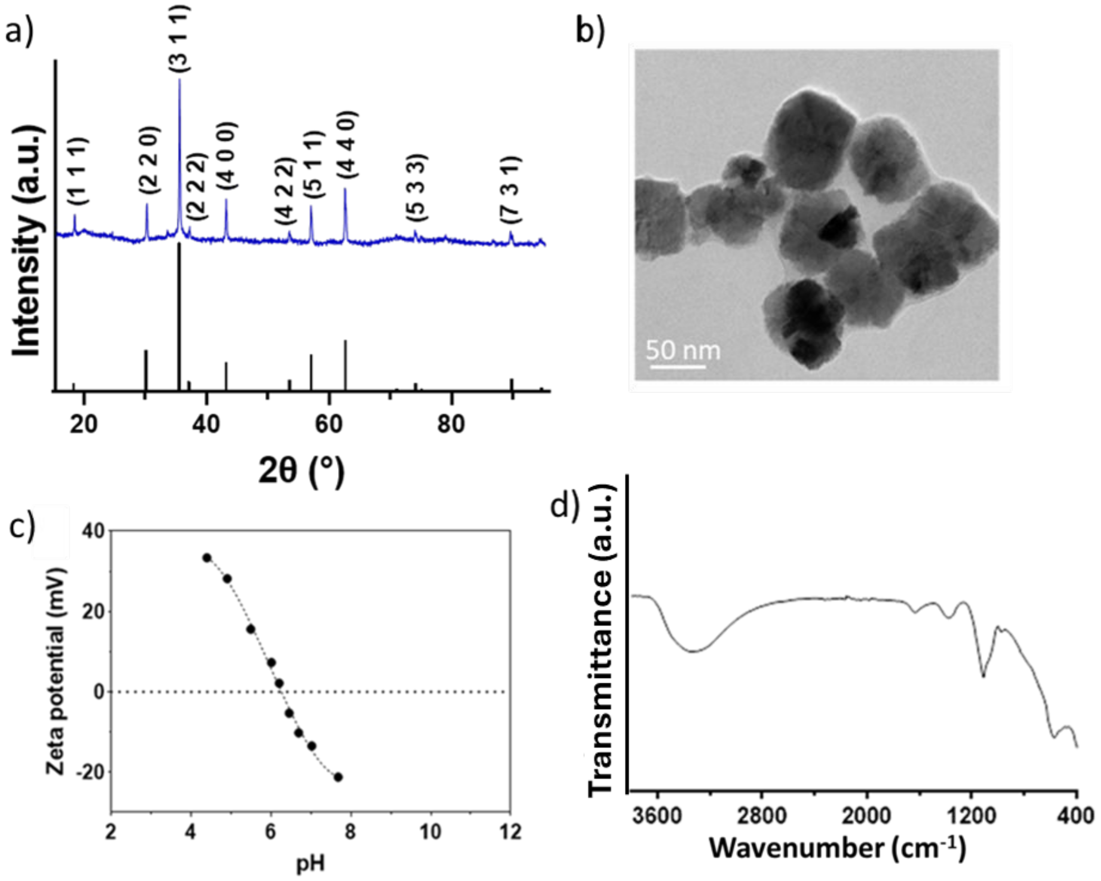

Figure 1SI – a) Comparison of experimental trace (blue) with the pattern (black bars) of $CoFe_2O_4$ nanoparticles (Card 04-010-1893 from database PDF-4+ International Center for Diffraction Data), b) Transmission Electron microscopy image of $CoFe_2O_4$ nanoparticles, c) Zeta potential of $CoFe_2O_4$ nanoparticles and d) FTIR-ATR of $CoFe_2O_4$ nanoparticles.

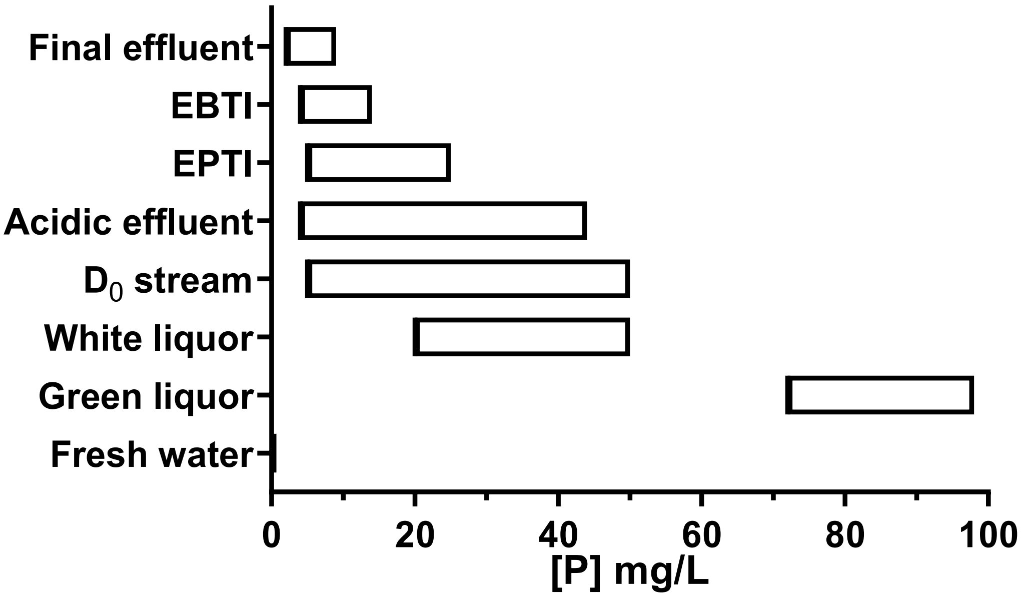

Figure 2SI – Distribution of P concentration across the various stages of a pulp mill production plant.

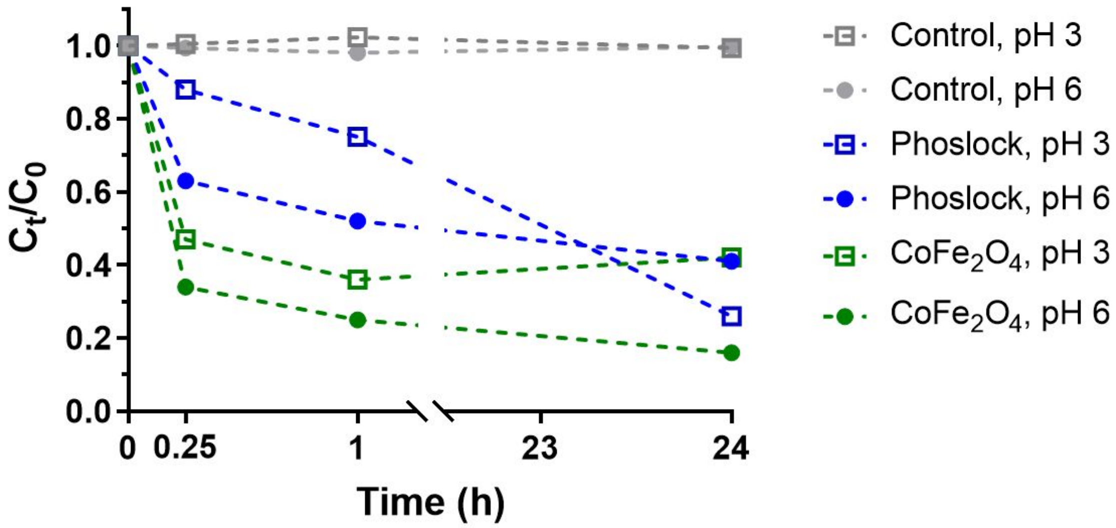

Figure 3SI – Comparison of the recovery efficiencies of P using cobalt ferrite nanoparticles and Phoslock (a commercial sorbent) from an acidic pulp stream with 25 mg P/L, 2 g/L of sorbent dose, 60 ºC, and pH 3 and 6.

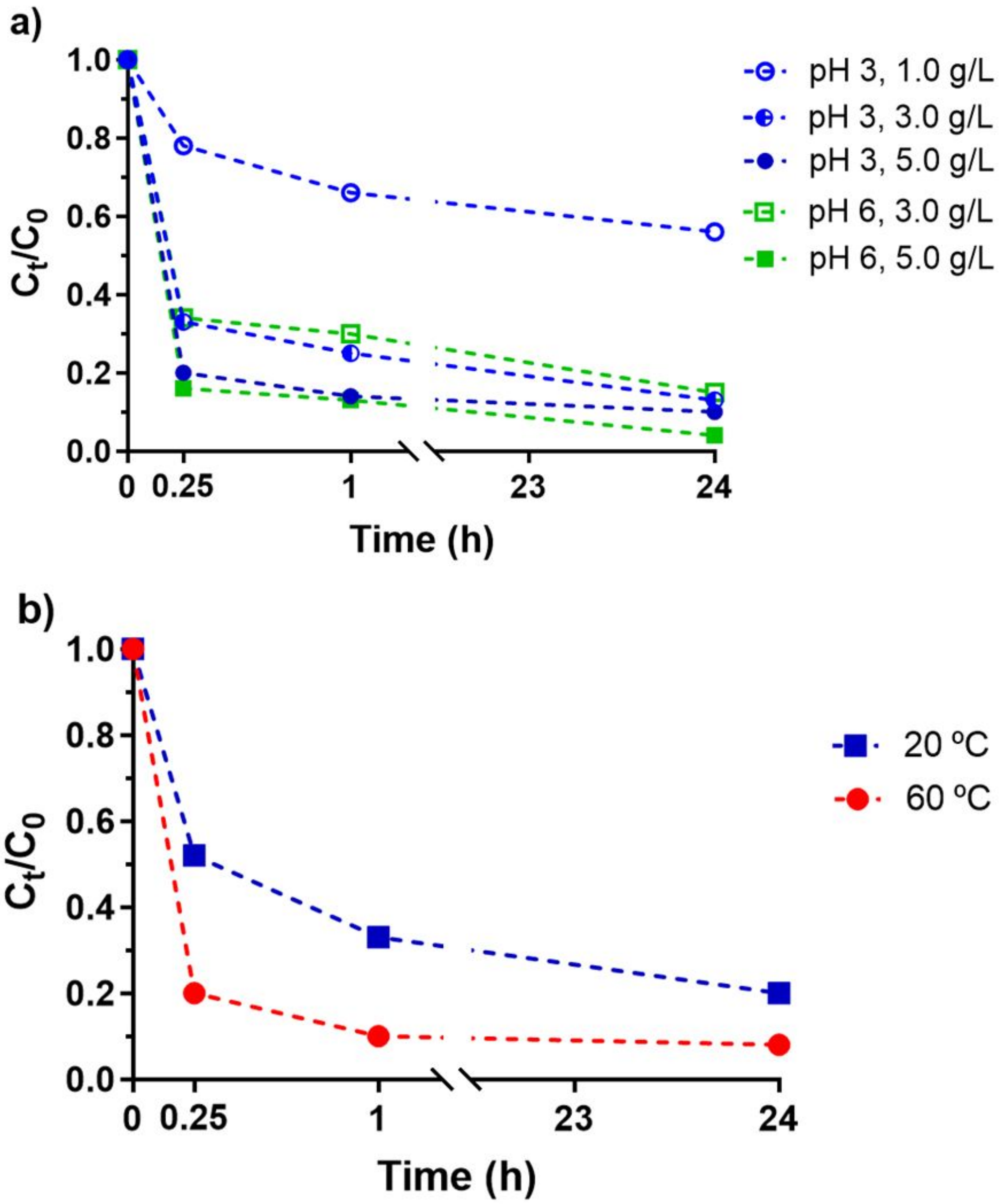

Figure 4SI – Variation of the normalized P concentrations over time for different experimental parameters using $CoFe_2O_4$ nanoparticles for an initial P concentration of 45 mg/L: a) Effect of sorbent dose and pH at 60 ºC; b) Effect of temperature with 5.0 g/L NPs at pH 3.

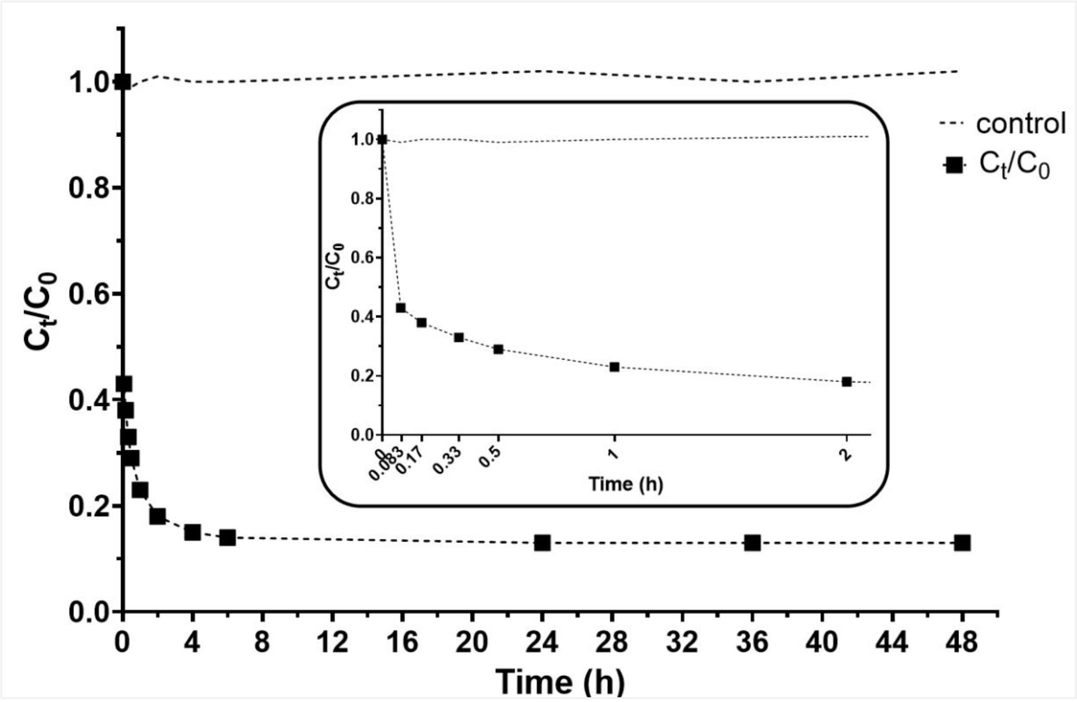

Figure 5SI – Variation of normalized P concentration over time using $CoFe_2O_4$ nanoparticles (1 g/L) at 60 ºC and pH 6 for an initial P concentration of 5 mg/L.

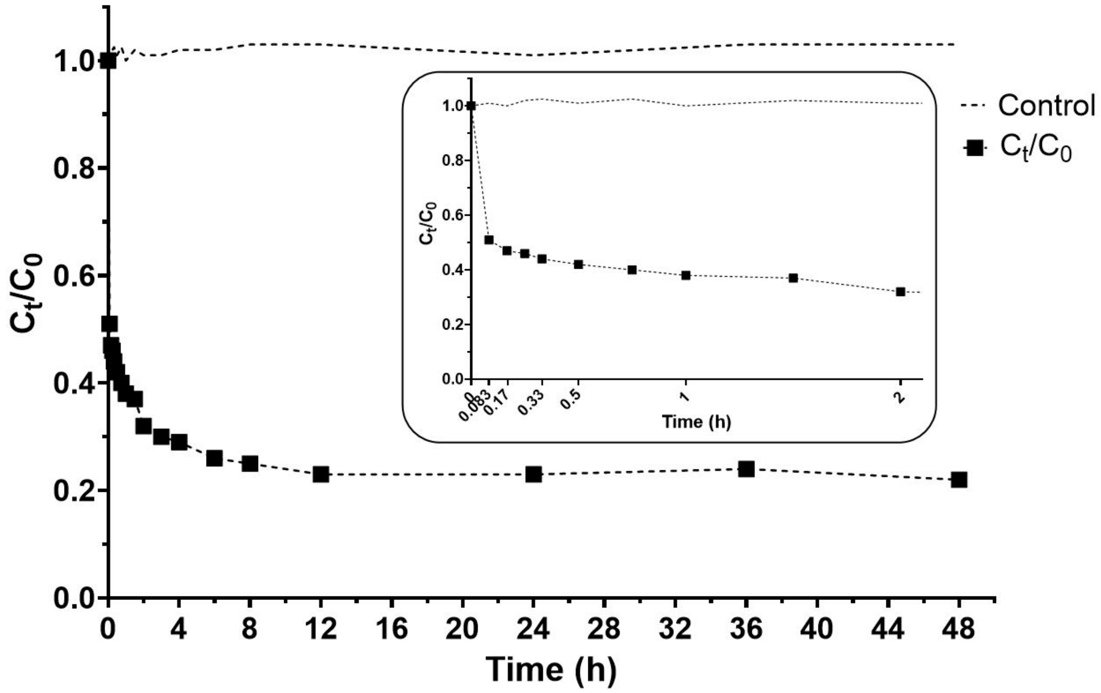

Figure 6SI – Variation of normalized P concentration over time using $CoFe_2O_4$ nanoparticles (2 g/L) at 60 ºC and pH 6 for an initial P concentration of 25 mg/L.

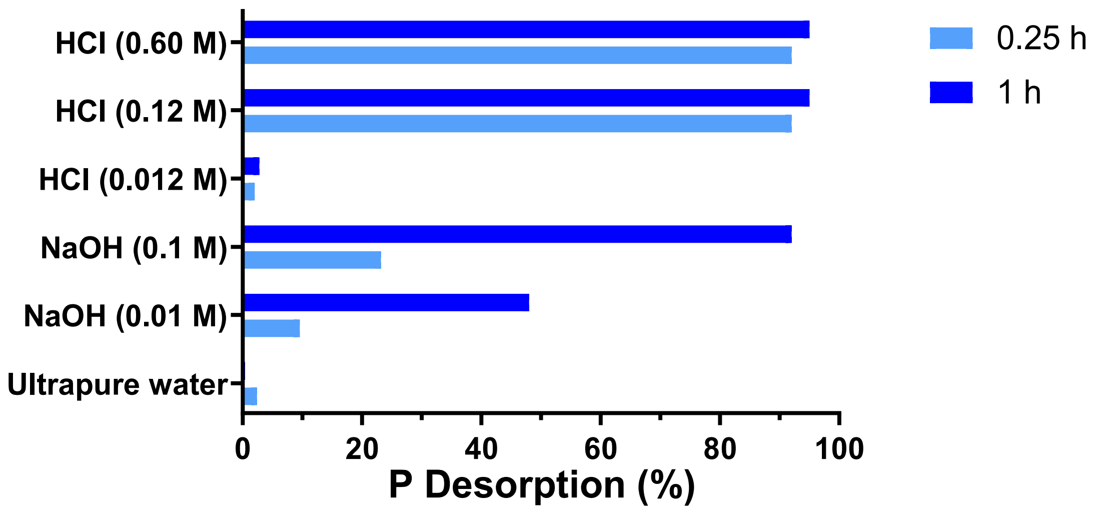

Figure 7SI – Desorption of P from cobalt ferrite NPs using acidic and basic solutions.

Equations

Kinetic models

Among the various kinetic models available in the literature, the kinetic models of pseudo 1st order (or model of Lagergren) and pseudo 2nd order are two simple mathematical models, widely used and known. These models are based on the arguments presented below.

The pseudo 1st order model, used to evaluate the sorption capacity of a given solid to the liquid phase, is expressed by the following equations (Lagergren, 1898):

$$\frac{dq_t}{\mathrm{dt}} = k_1(q_e - q_t) \xrightarrow[t=0(q_t=0)\ to\ t=t(q_t=q_t)]{integration} q_t = q_e(1 - e^{-k_1 t}) \quad \text{(I.2)}$$

where k_1 (h^{-1}) is the rate constant of the model. This model has the disadvantage of the failure to adjust to the experimental data is frequent, particularly when it tends to balance.

The pseudo 2nd order model, unlike the pseudo 1st order model, generally describe adequately the sorption process throughout all time period. The equation on which this model is based and the respective integrated form are as follows (Ho and McKay, 1999):

$$\frac{dq_t}{\mathrm{dt}} = k_2(q_e - q_t)^2 \xrightarrow[t=0(q_t=0)\ to\ t=t(q_t=q_t)]{integration} q_t = \frac{q_e^2 k_2 t}{1 + q_e k_2 t} \quad \text{(I.3)}$$

where k_2 (h^{-1}) is the kinetic constant of the model.

Another common kinetic model is Elovich's model and has been widely used to describe the sorption of gas onto solid systems (Low, 1960). However, more recently, this model has also been applied to the sorption processes of pollutants from aqueous solutions (Qiu et al., 2009). This model is based on the following equation:

$$\frac{dq_t}{\mathrm{dt}} = \alpha e^{-\beta q_t} \xrightarrow[t=0(q_t=0)\ to\ t=t(q_t=q_t)]{integration} q_t = \left(\frac{1}{\beta}\right)\ln\,(1 + \alpha\beta t) \quad \text{(I.4)}$$

where α and β are, respectively, the initial sorption rate, and the desorption constant.

References

Ho, Y.., McKay, G., 1999. Pseudo-second order model for sorption processes. Process Biochem. 34, 451–465.

Lagergren, S., 1898. Zur theorie der sogenannten adsorption geloster stoffe, 39th ed. ed.

Low, M.J.D., 1960. Kinetics of Chemisorption of Gases on Solids. Chem. Rev. 60, 267–312.

Qiu, H., Lv, L., Pan, B., Zhang, Q.Q., Zhang, W., Zhang, Q.Q., 2009. Critical review in adsorption kinetic models. J. Zhejiang Univ. Sci. A 10, 716–724.

Tables

Table 1SI. Parameters of the pseudo first-order, pseudo second-order, and Elovich kinetic models, associated to the sorption of phosphorus in a D_0 stream from kraft pulp mill with 5 mg P/L.

**$q_{t, exp}$ (mg/g)**		4.3
**Pseudo first-order model**	**$q_{e, cal}$ (mg/g)**	4.061
	**k_1 (min^{-1})**	11.58
	**R^2**	0.9274
**Pseudo second-order model**	**$q_{e, cal}$ (mg/g)**	4.226
	**k_2 (min^{-1})**	4.596
	**R^2**	0.9812
**Elovich model**	**B**	4.529
	**a**	4095686
	**R^2**	0.9728

Table 2SI. Parameters of the pseudo first-order, pseudo second-order, and Elovich kinetic models, associated to the sorption of phosphorus in a D_0 stream from kraft pulp mill with 25 mg P/L.

**$q_{t, exp}$ (mg/g)**		9.75
**Pseudo first-order model**	**$q_{e, cal}$ (mg/g)**	8.656
	**k_1 (min^{-1})**	9.130
	**R^2**	0.8404
**Pseudo second-order model**	**$q_{e, cal}$ (mg/g)**	9.075
	**k_2 (min^{-1})**	1.587
	**R^2**	0.9352
**Elovich model**	**B**	1.627
	**a**	196536
	**R^2**	0.9869
